# Role of microRNAs in the pathophysiology of sporadic colorectal cancer

**DOI:** 10.1186/1471-2458-12-S2-A22

**Published:** 2012-11-27

**Authors:** Fung Lin Yong, Chee Wei Law, Chee Woon Wang

**Affiliations:** 1 Department of Surgery, Faculty of Medicine, University of Malaya, 50603 Kuala Lumpur, Malaysia; 2 Department of Biochemistry, Faculty of Medicine, MAHSA University College, 59100 Kuala Lumpur, Malaysia

## Background

Colorectal cancer (CRC) is one of the leading causes of cancer-related mortality worldwide. Majority of the cases (~92%) are sporadic (nonhereditary), while the hereditary types constitute a lower percentage. The pathogenesis of sporadic CRC is heterogeneous and multi-factorial. In addition to environmental exposure, diet and lifestyle; accumulation of random somatic mutations also significantly affects the transcription of the genome and contributes to the carcinogenesis process. In recent years, microRNAs (miRNAs) have evolved as a unique class of endogenous regulators that offer great potential in the elucidation of cancer pathophysiology. The primary aim is to study the role of miRNAs as early biomarkers in sporadic CRC aetiology and pathogenesis.

## Materials and methods

Matched-pairs of 30 cancerous and non-cancerous tissues, 47 blood samples from sporadic CRC patients and 30 blood samples from healthy controls have been collected from the University of Malaya Medical Centre. Total RNA was extracted and profiled using Affymetrix GeneChip miRNA 2.0 microarray chips. The microarray results and predicted targets have also been analyzed using miRNA bioinformatic softwares.

## Results

A panel of significantly dysregulated miRNAs were identified (p < 0.05), namely miR-106a, -20a, -21, -223, -24 and -424. Based on TargetScan software, these miRNAs were found to participate in the regulation of keysignaling pathways in the adenoma-carcinoma sequences in CRC. Several predicted target genes involved were *APC*, *KRAS*, *PI3K*, *SMAD* and *MMPs*. These genes have been described to play crucial roles in inflammation, cell proliferation, apoptosis, angiogenesis, extracellular matrix remodelling and epithelial-mesenchymal transition.

## Conclusions

miRNAs are informative in shedding light on the molecular mechanisms underlying the pathogenesis of sporadic CRC and circulating blood miRNAs are reflective of those in tissues. Further studies into the blood miRNA profiles would elucidate their potential roles as novel noninvasive biomarkers in CRC.

